# Development of an Ethico-Legal Framework for Quality Improvement and Performance Management in Health Care: Protocol for a Qualitative Study

**DOI:** 10.2196/82167

**Published:** 2026-01-30

**Authors:** Kavisha Shah, Anna Janssen, Cameron Stewart, Tim Shaw, Ian Kerridge

**Affiliations:** 1 Faculty of Medicine and Health, University of Sydney, Level 2, Charles Perkins Centre, John Hopkins Drive, Sydney, NSW, 2050, Australia, +61 415 193 993; 2 Sydney Law School, The University of Sydney, Sydney, Australia; 3 Sydney Health Ethics, School of Public Health, Faculty of Medicine and Health, The University of Sydney, Sydney, Australia; 4 Haematology Department, Royal North Shore Hospital, Northern Sydney Local Health District, Sydney, Australia; 5 Philosophy Department, Macquarie University, Sydney, Australia

**Keywords:** health policy, ethics, privacy, transparency, procedural fairness, law, health data

## Abstract

**Background:**

The growing digitization of health data has expanded opportunities for professional learning and performance improvement. While they provide new means for improving the quality and safety of health care, these new capabilities for data analysis and performance monitoring come with risks and may exacerbate existing ethico-legal concerns about fairness, accountability, privacy, and more.

**Objective:**

This study aims to develop an ethico-legal framework for the evaluation of professional performance that is cognizant of these concerns and addresses the needs of relevant stakeholders. The study will assess the acceptability, comprehensiveness, and potential utility of the framework from the perspective of end users and subject matter experts.

**Methods:**

This study will use existing evidence on ethico-legal considerations surrounding secondary uses of health data for performance improvement and management to draft the framework. We will conduct 2 focus groups with end users (eg, health professionals and administrators) and subject matter experts (eg, clinical ethicists and legal practitioners). These focus groups will ask participants to reflect on the framework’s structure and comprehension, intended audience, comprehensiveness and relevance regarding ethical and legal principles, limitations, and utility and acceptability as a step-by-step guide. Study participants may also opt for one-on-one interviews for any reason. This feedback will be thematically analyzed using open coding and verified by an independent reviewer at the focus groups, followed by constant comparisons of feedback from this study to concepts and interrelationships in data previously collected.

**Results:**

Recruitment for this study is scheduled from August to December 2025. The analysis, compilation, and dissemination of higher-order themes, concepts, and outcomes is planned for after publication of this protocol, after each interview or focus group has been transcribed and coded line by line.

**Conclusions:**

This study seeks to create an actionable tool that is readily translatable to clinical practice in collaboration with end users and subject matter experts. The proposed methodology is a low-resource coapproach that could be iteratively refined to ensure that the proposed framework continues to support robust and efficient use of performance data while respecting the different contexts in which practice analytics may be delivered. This systematic approach to principle-led evaluation of performance and conduct could inform technology-neutral governance capable of addressing perennial concerns about fairness, privacy, and transparency when using health data for professional learning and performance management.

## Introduction

### Background

Reflective practice is foundational to professional practice in health care. It directly contributes to the quality and safety of patient care by encouraging health professionals to identify gaps in their knowledge, skill, or expertise and analyze failures, creating opportunities for professional development and growth [1]. Traditionally, health care has relied on self-nomination to identify deficiencies in professional performance [2], but this has limited effectiveness as health professionals frequently struggle to accurately assess their performance [3], identify their clinical or professional deficiencies or limitations, or use appropriate learning styles or tools to address knowledge or skill gaps [4]. In response, professional associations and regulatory bodies internationally have increasingly incorporated “practice analytics,” which broadly refers to the use of health data such as clinical outcomes and key performance indicators in performance evaluation as a condition of registration and/or accreditation [5]. While the use of practice analytics as a condition of registration is new, health data have previously been used to assess performance in the form of audit and feedback activities, clinical registries, and ad hoc data analysis [6-8].

While there are good reasons to support the use of practice analytics in these settings, we have collated a number of ethical concerns and medico-legal risks that have limited its application and uptake over the last 3 decades [9]. A scoping review undertaken by the authorship team over 2023 and 2024 in accordance with the PRISMA-ScR (Preferred Reporting Items for Systematic Reviews and Meta-Analyses extension for Scoping Reviews) [10] revealed that the lack of system integration and interoperability and the absence of well-validated, risk-adjusted outcome measures affected perceptions of fairness and justice when managing clinical issues and substandard performance [9]. More specifically, health practitioners were concerned about the risks of using crude measures that fail to capture the dynamic, complex, and heterogeneous nature of clinical practice to discipline individuals and introduce corrective action. The perceived unfairness of using these outcome measures to assess performance was also found to continue to impact trust in practice analytics given the potential consequences of disciplinary action to professional autonomy and reputations [9,11,12]. Beyond these immediate concerns about the use of health data in performance management, there appear to be increasing concerns about public disclosure of outcome measures by health administrators and regulators to the detriment of health practitioners and services [9]. Much of this hesitancy to have health care outcome measures publicly reported appears to relate to the possible misinterpretation and media sensationalization of data disclosed without proper deidentification and contextualization such that the broader clinical narrative remains underrepresented in publicly available information [9]. This reticence likely reflects emerging concerns about access to reflective statements in legal proceedings as punishment of open and honest practitioners [13] despite openness being an established aspect of good medical practice [14]. This treatment of practice reflections was also seen to encourage defensive practices when mistakes are made, thereby undermining professional accountability and compromising patient safety [15].

A review of the relevant literature also revealed that prevention of the escalation of defensive practice was seen to rely on networked forms of governance that meaningfully involved health practitioners in discussions about their performance and accounted for their preferences when deciding upon access to and disclosure of performance information [9]. In this regard, this review affirms the broader literature in that respect for and meaningful engagement of key stakeholders as valued and equal partners drives trust in programs that involve or affect these stakeholders, particularly when co-design and co-production of solutions occur [16]. This effect is particularly pronounced in the context of performance review as health practitioners are not only passive recipients of performance information but also essential to capturing actionable health data at the point of care and analyzing outcome measures using their specialized clinical expertise [17]. Therefore, delivering on the fundamental assurance of practice analytics as an initiative designed to improve patient safety and health care quality [18] depends on overcoming this key dependence and enhancing practitioner willingness to participate.

Our scoping review identified limited knowledge among key stakeholders on how ethical concepts are understood and applied and how legal risks are monitored and minimized in day-to-day practice so as to reduce the impact of perceived ethico-legal barriers on participation. This is reflected in the conflicting viewpoints on “transparency” among health practitioners, administrators, and the public in our scoping review, which we argued have to be reconciled to drive use of performance information [9]. To our knowledge, no study has comprehensively examined how this gap may be closed to guide secondary uses of health data for practice reflection and performance management. We present a research methodology for the development and refinement of an ethico-legal framework to encourage engagement with practice analytics in the workplace.

### Aim

This paper briefly outlines the protocol for the development of an ethico-legal framework and a strategy for exploration of the acceptability, comprehensiveness, and potential utility of this developed framework from the perspective of subject matter experts and end users.

## Methods

### Study Context

This study will involve qualitative semistructured interviews or focus groups conducted in Australia with subject matter experts and end users based in this country. This qualitative study will be reported in accordance with the Standards for Reporting Qualitative Research [19]. Key ethical concepts and medico-legal considerations used in the drafting of the framework have been modeled on feedback from health practitioners working in Australia and health services delivering care in Australia, in addition to the surrounding legal and regulatory context constructed by Australian governments, professional associations, and regulators.

### Development of an Ethico-Legal Framework

An ethico-legal framework for the secondary use of health data for quality improvement and performance management was developed using reviews of relevant literature, laws, governance, and policies governing secondary uses of health data in addition to interviews and focus groups with key stakeholder groups. The full methodologies and results of these reviews, focus groups, and interviews are to be reported elsewhere [9].

### Study Design

Reviews were conducted to document the clinical and regulatory rationale for using health data as performance feedback for workforce evaluation and management. These reviews sought to identify key ethico-legal principles used to rationalize the implementation and use of practice analytics for practice reflection and performance management and document the ways in which it is currently operationalized in clinical practice internationally. This included a scoping review of academic manuscripts and conference proceedings available online from the first published materials available on leading medicine and health databases [9], a policy review of relevant guidelines and standards, and a legislative review of applicable laws and regulations. Semistructured qualitative interviews were then conducted with 14 key stakeholders to identify ethico-legal principles that guide implementation of practice analytics, influence professional attitudes toward and engagement with practice analytics in performance evaluation, and/or affect engagement with and impact of the registration requirement positively and negatively. Key stakeholders approached and interviewed included health professionals eligible for registration in Australia; health administrators; and representatives of regulatory bodies, professional associations, and key consumer groups. A key finding from these reviews and interviews was a focus on consistency in the regulation and governance of practice analytics to alleviate worries and a preference for a gradated multistep process for the identification, analysis, and management of clinical variance.

This gradated approach was adapted in the framework as a step-by-step guide consisting of 5 conditional steps with embedded prompts to structure reflections and deliberations on professional performance that use health data as a source of evidence. Descriptions of ethico-legal principles were also extracted and synthesized from these reviews and interviews into a short paragraph no longer than 100 words to enable due consideration of principles during practice reflection and performance management. Many of the prompts explicitly framed ethico-legal principles as questions to encourage discussion on whether these principles are relevant to the reflection and performance review and should be applied. For example, the prompt for trust is “Does the performance/conduct impact public confidence in the quality and safety of healthcare?” and the prompt for honesty is “Were professional responsibilities undertaken honestly, in good faith and with a reasonable degree of care? Was there timely disclosure of unintended consequences to relevant parties?”

The authorship team reviewed this framework, with minor revisions made for comprehension, readability, and clarity, such as removing duplicated prompts, adopting plain language, and including additional features to reduce user error. Multimedia Appendix 1 presents the first draft of the framework for presentation to subject matter experts and end users as outlined in this protocol.

### Refining an Ethico-Legal Framework

Two focus groups lasting 1 to 2 hours will be conducted with 5 to 10 purposefully selected participants. Participants will be asked to reflect on six key considerations: (1) framework structure and comprehension, (2) intended audience, (3) comprehensiveness and descriptions of ethical and legal principles, (4) relevance of principles to professional performance and practice more broadly, (5) acceptability and utility of the step-by-step guide, and (6) limitations of the framework. Participants will then be asked to briefly reflect on the potential utility of the framework and when the framework would be most useful in practice through reflections on past experiences or creation of clinical vignettes or case studies.

### Participants

#### Inclusion and Exclusion Criteria

Participants are eligible to take part in the study if they have subject matter expertise relevant to the framework or could be end users of the framework. Potential end users include members of the health workforce involved in performance evaluation, review, and monitoring. This could include registered health professionals, health administrators, and executive staff (eg, directors of clinical governance) working in Australia or supporting the delivery of health services in Australia. Students will be ineligible to take part in the study as students are not obligated to review performance and measure outcomes related to their performance as a condition of their registration or practice [5]. Individuals will also be eligible to take part if they are policymakers or representatives from professional associations and regulatory bodies involved in the enforcement of standards mandating the implementation of practice analytics or outcome measurement in Australia. Subject matter experts include individuals with expertise in health law, clinical ethics, and bioethics due to their specialized knowledge, training, or experience in these fields. Individuals with expertise in health law will be required to have an understanding of Australian statutes, common or judge made, and regulations. Any participant with no connection to health service delivery in Australia or knowledge of the Australian legal and regulatory context will be excluded from eligibility.

#### Recruitment

Eligible participants and their contact details will be compiled by the authorship team from publicly available information and their personal and professional networks. Each participant will be contacted via email by the lead investigator with a link to the participant information statement and consent form. This email will indicate how this individual was identified as an eligible participant and provide a brief summary of the study and the option to participate in a one-on-one interview for any reason. Participants will also be made aware of their option to contact a member of the research team to discuss the study before consenting to take part. Participants can self-select for an in-person, online, or hybrid discussion in addition to either an interview or focus group, with in-person interviews and focus groups to be conducted at the University of Sydney. Participants who do agree to take part will be asked to forward information about the study to others who may be interested in participation to assist with snowball recruitment after confirmation with the research team.

### Data Collection Procedures

A semistructured guide (Multimedia Appendix 2) was developed consisting of open-ended questions and prompts stratified against 1 or more of the 6 considerations or topics: (1) framework structure and comprehension, (2) intended audience, (3) comprehensiveness and descriptions of ethical and legal principles, (4) relevance of principles to professional performance and practice more broadly, (5) acceptability and utility of the step-by-step guide, and (6) limitations of the framework. Data collection will continue until at least 5 or a maximum of 10 individuals belonging to both stakeholder groups (subject matter experts and end users) have taken part in an interview or focus group. Depending on the rate of recruitment and level of expressed interest in the study, we may keep recruitment open until the minimum number of participants has been reached or cap recruitment at the maximum number of participants.

Focus groups or interviews will be conducted in person and/or online using the Microsoft Teams or Zoom (Zoom Video Communications) videoconferencing platforms depending on participants’ preferences. Focus groups or interviews will be recorded using the built-in recording capabilities of the platforms and transcribed verbatim by the facilitator. These recordings and transcripts will be downloaded onto a secure, enterprise-grade network-attached storage hosted by the University of Sydney accessible only to the lead investigator and facilitator. These transcripts will then be deidentified for analysis by the facilitator and members of the authorship team.

### Data Analysis

The framework method for qualitative analysis [20] will be adapted to make iterative refinements by linking data obtained in this study to themes and categories obtained from previous data used to draft the framework [21]. This analytical method is favored as it does not prescribe to a theoretical or epistemological approach but, rather, enables the interpretation of study data against predefined topics, as undertaken in this study with the identification of 6 key considerations for feedback on the framework [20]. This methodology also enables us to move along the inductive-deductive continuum to link concepts and themes from this study to those from prior studies while allowing for unexpected responses and temporal and contextually situated feedback from participants in this study [21].

This analysis will commence with immersion in the raw data by the facilitator who will read and reread the transcripts shortly after completion of the interviews or focus groups. This process of familiarization will allow for timely reporting of emerging themes and facilitation challenges to the broader authorship team for review and discussion. These discussions may inform potential revisions to the framework and/or semistructured interview guide to allow the research team to remain agile to evolving study findings and minimize the risk of outdated or repetitive research findings [22]. This preliminary reporting will be followed by open coding, where each interview or focus group transcript will be coded line by line by one author. This open coding will take place in Microsoft Word, with a new comment generated for each line of text. This open coding will then be drawn together based on relationships between line codes and higher-order concepts and outcomes already identified in prior studies or the framework itself, or constant comparison. New concepts and interrelationships will be affirmed by the wider authorship team. A member of the authorship team will be invited to be an independent reviewer in the focus groups to maintain a contemporaneous record of initial impressions, emerging concepts, and theoretical connection. These analytical insights will be combined with feedback from this reviewer to enhance the credibility of the research findings and minimize actual or apprehended bias of this authorship team given our involvement in the development of the draft framework. This reviewer will also be asked to examine the transcripts, codes, and concepts to identify new or miscategorized concepts after completion of data analysis by the research team. These groupings will then be mapped against the predefined considerations [22], namely, the acceptability, utility, and comprehensiveness of the framework as a tool for practice reflection and performance management.

### Ethical Considerations

Ethics approval for this study has been received from the University of Sydney Human Research Ethics Committee (2022/643). Each eligible participant will be sent an email invitation to take part in the study. This invitation will contain the participant information statement and participant consent form with a short summary of the study objectives. The focus group or interview will be scheduled with the return of a completed participant consent form. After completion of the focus group or interview, recordings and transcripts will only be accessible to the facilitator, who will then deidentify the information to protect the privacy and confidentiality of participants before analysis.

## Results

Enrollment of study participants started in August 2025 and is expected to end in December 2025. An in-depth analysis has commenced in late September 2025 and will likely be completed in early January 2026, as will collation and description of relevant themes for broader dissemination in conference presentations, research seminars, doctoral theses, and journal articles. At manuscript submission, we had conducted 2 focus groups and 6 interviews from October to December 2025. A total of 15 participants were interviewed. Data analysis is underway, with coding and thematic mapping completed for 1 focus group and 2 interviews in November 2025. The remaining data will be analyzed in February 2026 and prepared for publication in March 2026.

## Discussion

This research protocol has been designed to enable the development and refinement of an ethico-legal framework to support decision-making related to the secondary use of health data for quality improvement and performance management. The goal of such a framework is to address real and perceived ethico-legal concerns that appear to determine attitudes toward and engagement with the use of practice analytics for practice reflection and performance management [9].

### Potential Findings

We expect that this framework will be well received by end users as a useful tool for health practitioners and administrators, but how best to introduce and scale this framework across medical specialties and services, particularly the proposed format and length of the step-by-step guide, is unclear. Possible suggestions may include the development of an electronic tool to reduce the perceived complexity of the framework, which has previously been identified as a critical feature of successful audit and feedback systems and practice change initiatives such as reminders at the point of care [23]. These considerations will likely be critical to widespread uptake of the framework as they will reduce actual or perceived barriers to accessing and applying this tool in day-to-day clinical practice. The findings of the proposed study could have significant implications for quality improvement initiatives and performance management in health care. This study could help focus the conversation away from feasibility and effectiveness testing of each new technology or technique for data collection onto knowledge mobilization and the socialization of performance information using a consistent, harmonized approach.

### Comparison With Prior Work

This distinction is increasingly important as barriers to the use of outcome measures and other performance measures appear to depend on data contextualization either through appropriate risk adjustment or full and meaningful engagement of medical practitioners in discussions about their performance [15]. Consequently, a key strength of the proposed study is the fact that the development process involves the systematic identification of ethico-legal concerns regarding practice analytics from a detailed review of the relevant literature and in-depth interviews with relevant subject matter experts and key stakeholders engaged in performance management or subject to it. The collection of in-depth qualitative feedback from key stakeholders has also enabled the research team to tailor research outputs to the local context, including the decision to develop this framework, and consider sociocultural influences and the broader regulatory landscape. The findings of these preceding studies have identified the importance of robust governance and data stewardship for promoting stakeholder engagement with their performance data such that the framework, if well received, will help close an evidence-practice gap.

Furthermore, many of the concerns about privacy and consent appear to be contingent on robust governance over the collection, use, and disclosure of personal and health information, as opposed to focusing on the technical infrastructure used to collect this information alone [9]. The former approach could be taken to inform security assessments of the latter such that technology-specific research may preclude in-depth evaluation of social, cultural, and political influences over medical decision-making and health service planning [24]. Favoring a technology-neutral approach also mimics the prioritization of technology-neutral regulation in the broader legal and regulatory landscape whereby rules are made deliberately flexible to be applicable irrespective of the technology used to reduce compliance costs and burdens to citizens [25], or end users in this case. We acknowledge that this strength may be unique to the Australian context and other jurisdictions where technology-neutral policymaking is prioritized and serves as a limitation on the generalizability of the research outputs. However, this methodological approach does allow for iterative refinements such that outcomes related to earlier studies could be updated periodically as new evidence emerges and repeated with different audiences over time to respond to technological advancements and dynamic contexts.

While many of the studies included in our scoping review identified key ethico-legal considerations surrounding practice analytics [9], principle-focused evaluations are a relatively new program of work in medical education and professional development [26]. This does not mean that guiding principles are not implemented in practice, merely that the nature of their incorporation is highly varied and often dependent on workplace champions to model and disperse a culture of value-led learning and participation [27]. The proposed study seeks to explore a different coapproach to how principles could be actioned in performance evaluations as a systematic exchange of information based on an agreed set of relevant and acceptable ethico-legal values compared to the potential for organic expression in routinized ways of working [28] and whether this systemic approach is capable of driving engagement with performance feedback and practice change. In doing so, the proposed study may be able to help realize the benefits of explicit deliberation on principles when difficult decisions need to be made about professional performance and conduct. This could include procedural improvements, from providing direction and guidance on conducting fair reviews to designing sanctions that are proportionate to the departure from the accepted standard [26], in addition to protecting against arbitrary decision-making through full and meaningful consideration of relevant factors [29].

### Limitations

Realizing these hypothesized benefits will depend on effectiveness testing across health care settings (ie, inpatient and outpatient, chronic and acute care, or public and private), research that is beyond the scope of this protocol and an inherent limitation on the impacts of the proposed study. We also note that the use of purposive and snowball sampling may result in the recruitment of highly motivated individuals invested in the successful implementation of the framework. This recruitment and response bias may obscure potential limitations of the framework from the perspective of end users, particularly those who may find principle-led evaluation desirable but not strictly necessary in routine practice.

### Conclusions

This study aims to refine the drafted framework with new information about its value and acceptability from implementers of practice analytics. This novel information will inform translation of the resource into day-to-day practice and possibly contribute to improvements in the safety and quality of health services provision.

## Supplementary material

10.2196/82167 Multimedia Appendix 1 First draft of the ethico-legal framework.

10.2196/82167 Multimedia Appendix 2 Interview and focus group guide.

10.2196/82167 Checklist 1 SRQR checklist.

## Abbreviations

PRISMA-ScR: Preferred Reporting Items for Systematic Reviews and Meta-Analyses extension for Scoping Reviews
SRQR: Standards for Reporting Qualitative Research
