# Supplementary material for: Development of an Ethico-Legal Framework for Quality Improvement and Performance Management in Health Care: Protocol for a Qualitative Study
Source: JMIR Res Protoc. 2026 Jan 30;15:e82167. doi: 10.2196/82167 (PMC12857889; doi:10.2196/82167)
Supplement: Multimedia Appendix 1 [file resprot-v15-e82167-s001.docx]

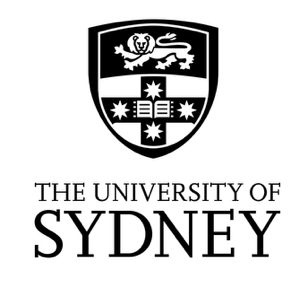

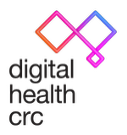


**Step-by-step guide to support performance review and reflection**

September 2025

NOTE: *This is a draft framework under review. Please do not share a copy of this framework without express permission from the drafters. Please contact Kavisha Shah (*[*kavisha.shah@sydney.edu.au*](mailto:kavisha.shah@sydney.edu.au)*) if you have any questions or concerns.*

Table of Contents

[Table of Contents 2](#_Toc207480505)

[Background 3](#_Toc207480506)

[Purpose 3](#_Toc207480507)

[Scope and Intended Audience 3](#_Toc207480508)

[Structure of the Framework 3](#_Toc207480509)

[Step-by-Step Guide 4](#_Toc207480510)

[Identify stakeholders and collect evidence 4](#_Toc207480511)

[Review and evaluate performance 4](#_Toc207480512)

[Specify the issue or concern 5](#_Toc207480513)

[Propose actions and give reasons 6](#_Toc207480514)

[Confirm decision 7](#_Toc207480515)

[Appendix A: Relevant Resources – Professional Standards 8](#_Toc207480516)

[Appendix B: Procedural and Substantive Ethical Values 10](#_Toc207480517)

[Appendix C: Legal Explainers 12](#_Toc207480518)

[Identify stakeholders and collect information/evidence 12](#_Toc207480519)

[Review and Evaluate Performance 14](#_Toc207480520)

[Specify the issue or concern 14](#_Toc207480521)

[Propose actions and give reasons 15](#_Toc207480522)

[References 16](#_Toc207480523)

Background

Purpose

This Framework is a step-by-step guide to support performance appraisal and review as a standalone activity and as part of continuous professional development, complaints management, incident review, case conferences and more. The Framework is designed to support:

- Robust and critical appraisal of performance
- Identification of key stakeholders and collection of relevant information/evidence
- Reflection on broadly applied ethical values (procedural/substantive)
- Due consideration of the surrounding legal and regulatory context.

The Framework is also tailored to guide users on how best to use health data as a source of information/evidence in your decision-making (“practice analytics”).

Scope and Intended Audience

The Framework is designed principally for medical practitioners, but it may also assist with practice reflection by, and performance review and monitoring of, other health practitioners. If used for non-medical practitioners, you may need to consider profession-specific policies and advice. The Framework is also not a substitute for legal advice. It should be used alongside applicable policies and procedures and verified against relevant laws and regulations in your jurisdiction.

Structure of the Framework

The Framework consists of five steps with embedded prompts to support robust and critical appraisal of performance. Some steps are conditional, and not all reflections/reviews will require you to complete each step or answer each prompt. You may also spend more or less time on each step depending on the goal of each reflection/review. This step-by-step guide is accompanied by three appendices:

1. **Appendix A: Relevant Resources – Professional Standards***.* This Appendix provides links to codes of conduct, professional standards and other guidelines commonly relied upon by judicial and administrative bodies to identify the relevant professional standard.
2. **Appendix B: Procedural and Substantive Values***.* This Appendix identifies key ethical values relevant to the review of professional performance. Not all values will be equally relevant, and you may need to use your judgment as to which values should be applied in your reflection/review.
3. **Appendix C: Legal Explainers***.* This Appendix presents a high-level summary of key legal considerations commonly applied in administrative and judicial proceedings assessing professional performance. These explainers are not a substitute for legal advice.

| **Goal of the reflection/review** | **Proposed Steps** | **Types of activities** |
| --- | --- | --- |
| Regular reflection of your performance/conduct. Improvements may be possible but are not essential for patient safety. | Complete Steps 1 and 2.  Skip ahead to Steps 4 and 5. | Practice reflection  Continuous professional development  Case conferences |
| You may have identified a potential departure from accepted professional standards or clinical guidelines, or there is some concern for patient safety. | Complete Steps 1 and 2.  Focus on Step 3.  Conclude with Steps 4 and 5. | Complaint or concern  Clinical incident  Serious adverse event review |

Step-by-Step Guide

| **BEFORE** *starting the reflection/review, consider:* | | | |
| --- | --- | --- | --- |
| **1** | |  | Identify stakeholders and collect evidence |
|  | | | |
| **What is the goal of this reflection/review?**   - Is it: *peer review, performance appraisal, professional development, case conferences, morbidity and mortality meeting, complaint or concern, clinical incident or serious adverse event review etc.* - Depending on the goal of this reflection/review:   - Should peers or colleagues be a part of the reflection/review?   - Should patients, families and/or consumer advocates be a part of the reflection/review?   - Should you invite others with clinical responsibilities for governance, health services and more?   - Should others be made aware of this reflection/review?   - How should these individuals or groups be engaged? Do they need to be informed, consulted or involved? - Who will be impacted by this reflection/review? E.g., *oneself, colleagues, current/future patients* | | | |
|  | | | |
| **What information/evidence do you need for this reflection/review?** | | | |
| SOURCES | - Health data e.g., *audits, service and prescribing history, clinical outcomes, patient-reported measures, clinical notes, medical records, clinical registries* - Peer reports and external expert reviews - Patient feedback e.g., *compliments and complaints* - Professional standards, codes of conduct and employment codes - Published data, clinical guidelines and epidemiological studies | | |
| CONTEXT | What are the strengths and limitations of this information/evidence? *Consider:*   - Is this information/evidence relevant to the goal of the reflection/review? Is it high-quality? Is it representative of the full circumstances of the case? - Is the health data adjusted for patient condition(s) and/or population(s)? - If others should be involved, or are likely to be impacted, would they accept the available information/evidence? If so, how would these individuals evaluate this information? - Do you need more information to make this decision? | | |
|  | | | |

| **DURING** *the reflection/review, consider:* | | | | | |
| --- | --- | --- | --- | --- | --- |
| **2** | |  | | Review and evaluate performance | |
|  | | | | | |
| **What are the relevant professional standards and clinical guidelines?** | | | | | |
|  | | | | | |
| **What are the relevant ethical values and implications of this review of performance?**  *Depending on the goal of this reflection/review, consider whether the following values apply:* | | | | | |
| **Trust** | | | | | Does the performance/conduct impact public confidence in the quality and safety of healthcare? |
| **Honesty** | | | | | Were professional responsibilities undertaken honestly, in good faith and with a reasonable degree of care? Was there timely disclosure of unintended consequences to relevant parties? |
| **Integrity** | | | | | Did the conduct involve or give rise to an actual or perceived conflict of interest? If so, what steps were taken to minimise this conflict of interest? |
| **Respect** | | | | | Was the performance/conduct respectful of others? |
| **Equity** | | | | | Was there any discrimination on the basis of age, sex, gender, disability, race and other personal characteristics? Should reasonable accommodations have been made to improve service delivery or patient outcomes? |
| **Justice** | | | | | Does this reflection/review give due consideration to relevant interpersonal, organisational and system factors that contribute to performance/conduct without bias or prejudice? |
| **Engagement & Reasonableness** | | | | | Have key stakeholders been given the opportunity to review and respond to information/evidence, or informed about progress/delays? Would a reasonable person in your shoes find this reflection/review fair? |
| **Consistency** | | | | | Have you been in a similar situation before, and how did you respond? Should a similar or different approach be taken here? |
| **Efficiency** | | | | | Is this reflection/review being conducted in a timely manner? Have key stakeholders been informed about review progress and possible delays? |
| **Privacy** | | | | | Are there real/perceived concerns regarding privacy and confidentiality? If so, what should be done to minimise these risks? |
| **Openness,**  **Transparency &**  **Stewardship** | | | | | What are the procedures for the collection, use, storage and disclosure of information/evidence? Have key stakeholders been informed of the benefit and risks? |
|  | | | | | |
| **Is it possible that there has been a departure from accepted professional standards?**  If yes, **go to Step 3.** If no, **go to Step 4.** | | | | | |
|  |  | |  | | |

| **IF** *you are conducting a review* **OR** *your reflection may indicate a* ***potential*** *departure, consider:* | | | | | |
| --- | --- | --- | --- | --- | --- |
| **3** | | |  | | Specify the issue or concern |
|  | | | | | |
| **Which professional standards or clinical guidelines are relevant to the potential departure?**  *Ask yourself:*   - What level of training and experience is possessed by the health professional? What standard of knowledge or care would be expected of a health professional with that level of training and experience?^[1]^ | | | | | |
|  | | | | | |
| HARM | **What is the potential risk of harm?**  *Ask yourself:*   - Is the risk of harm far-fetched or fanciful? Or is it real and foreseeable?^[2]^ - What is the type of harm? Is it physical, psychological and/or economic? | | | | |
| MINIMISATION | - How severe or significant is the harm? - What are the ethical implications and unintended consequences of the departure? - Who (and how many) stakeholders will be affected by the departure? Could these key stakeholders have done anything to prevent or lessen the risk of harm? - Is this a one-off incident or a repeated issue? - Have precautions or corrective action been taken to address the issue or concern? Is this precaution or corrective action appropriate and adapted to the departure? - Do you need more information? Return to **Step 1.**   *If there is a substantial risk of harm to the public, you may have to consider making a notification to your health complaints entity about this departure.* | | | | |
|  | | | | | |
| **How significant is the potential departure from accepted standards and clinical guidelines?**  *Ask yourself:*   - Was the knowledge or care demonstrated here below the relevant standard or outside of accepted guidelines for performance/conduct?^[1]^ - How serious is this potential departure from accepted professional standards? Would it be obvious to a health practitioner in the shoes of the person responsible for the potential departure? - Do you need more information? Return to **Step 1.**   *If it is more than a slight or moderate departure, you may have to consider a notification to your health complaints entity about this act/omission.* | | | | | |
|  | |  | |  | |

| **AFTER** *a reflection/review, consider:* | | | | | |
| --- | --- | --- | --- | --- | --- |
| **4** | | |  | Propose actions and give reasons | |
|  | | | | | |
| **What are the areas of improvement(s)?**  *Ask yourself:*   - What was done well? What could have been done better? - If there was a departure, who holds clinical responsibility, and who is responsible for monitoring performance/conduct to prevent similar departures? What steps can be taken to collaborate with these stakeholders? - What additional considerations should have been taken into account by the decision-maker(s)? - Do you need more information? Return to **Step 1.** | | | | | |
|  | | | | | |
| **Propose an action.**  *Ask yourself:*   - What are the intended and unintended consequences of the proposed actions(s) for the patient, the health practitioner and the health system? - What resources do you need to implement the proposal, and monitor its implementation? Who do you need to involve to ensure successful uptake of the proposed action(s)? - If there was a departure, how could you close the gap between the departure and accepted professional standards? What are some alternative actions that could have been taken? | | | | | |
| **Trust** | | | | | What action(s) are necessary to maintain public confidence in the quality and safety of health care? |
| **Public benefit** | | | | | What actions(s) should be taken to maximise benefits for the health and wellbeing of the broader patient population(s) and/or public? |
| **Proportionality & Autonomy** | | | | | Is the proposed action(s) appropriate and adapted to the identified area(s) of improvement? Does it constitute the minimum intrusion onto current practices and the free exercise of clinical judgment needed to address an issue or concern? |
| **Accountability** | | | | | Is there a clear assignment of core responsibilities, duties and tasks for performance/conduct, reflection/reviews and proposed actions? How will these be enforced? |
| **Solidarity** | | | | | Do the proposed action(s) involve an equitable distribution of benefits and burdens across key stakeholders *e.g., patients, peers or colleagues, health system, public*? How could a more fair and equitable distribution be achieved? |
| **Consistency** | | | | | Are there any similarities or differences to past reflections/review? What were the proposed actions in reflections/reviews with similar circumstances or concerns? |
|  |  |  | | | |

| **5** | |  | Confirm decision | |
| --- | --- | --- | --- | --- |
|  | | | | |
| **Document your decision.**  *Provide a written summary of* the proposed action, key outcome(s) and how proposed actions will be monitored and by whom. Give reasons for the proposed action, ethical values that support this action and evidence used when making the decision. | | | | |
|  | | | | |
| **Set a date and time for your next reflection.** | | | |  |
|  |  |  | | |

Appendix A: Relevant Resources – Professional Standards

Professional standards are established through codes of conduct, best practice guidelines for treatment and care, and more often than not widely accepted practices by the health profession. This list presents a non-exhaustive summary of standards, defined as those most cited in health policies across public health services, regulatory bodies or professional associations and/or commonly applied in proceedings before health complaint entities, civil and administrative tribunals and courts.

**Codes of Conduct and Professional Standards**

- [*Good Medical Practice: A Code of Conduct for Doctors in Australia*](https://www.medicalboard.gov.au/Codes-Guidelines-Policies/Code-of-conduct.aspx)*.* The principles and guidance on professional conduct outlined in this code are some of the most commonly applied standards in proceedings, and it is used to establish what is expected of medical practitioners in most circumstances, and whether or not there has been a departure from these expectations. Key sections with respect to practice analytics include sections 3.2. Good patient care, 4.3 Effective communication, 9.2 Continuing professional development and 10.5 Medical records.
- [Australia Medical Association. Code of Ethics. Revised 2016.](https://www.ama.com.au/articles/code-ethics-2004-editorially-revised-2006-revised-2016) This code is referred to understand the broader community expectations of professional conduct when interacting with patients or colleagues as well as making representations to the public in their professional capacity.
- [Australian Commission on Safety and Quality in Health Care Clinical Governance Standard](https://www.safetyandquality.gov.au/standards/nsqhs-standards/clinical-governance/clinical-governance-standard)
- [Australian Commission on Safety and Quality in Health Care. The Australian Open Disclosure Framework](https://www.safetyandquality.gov.au/our-work/open-disclosure/the-open-disclosure-framework) to guide disclosure of performance that falls below the accepted standard.
- It is also strongly encouraged to refer to the respective codes released by professional associations: [ANZCA Supporting Professionalism and Performance](chrome-extension://efaidnbmnnnibpcajpcglclefindmkaj/https:/www.anzca.edu.au/getContentAsset/31320f5b-4fa5-4e12-81f3-29f1d1679b64/80feb437-d24d-46b8-a858-4a2a28b9b970/ANZCA-Professionalism-Performance-Guide_2024.pdf?language=en), [RANZCP Code of Ethics](chrome-extension://efaidnbmnnnibpcajpcglclefindmkaj/https:/www.ranzcp.org/getmedia/2e090981-cdd2-4dee-a317-f8718bc7dc47/ranzcp-code-of-ethics.pdf), [RACS Surgical Competence and Performance](chrome-extension://efaidnbmnnnibpcajpcglclefindmkaj/https:/www.surgeons.org/-/media/Project/RACS/surgeons-org/files/reports-guidelines-publications/manuals-guidelines/surgical-competence-and-performance-framework_final.pdf), [RACP Professional Practice Framework](https://www.racp.edu.au/fellows/professional-practice-framework) and more.

**Employment Contracts and the Incorporation of Workplace Policies**

Terms and conditions of practice set out in employment contracts or contractual agreements for visiting medical officers may require compliance with written policies of the respective health service. The incorporation of these policies and procedures into any employment contract should be properly assessed as any breach of the policies may constitute a breach of the contract and result in an award of damages against the practitioner or health service. This is particularly relevant with respect to professional performance as disciplinary procedures incorporated into contracts must be strictly adhered to, and any updates must be properly acknowledged and accepted by the medical practitioner.

**Peer professional practice**

Peer professional practice is raised as a common defence in medical litigation. It states that a health practitioner is not negligent in providing a health service if the health practitioner acted in a manner that is widely accepted, by a significant number of practitioners in the field, as competent practice in these circumstances.^[3]^ Peer professional opinions may also indicate when departures from professional codes or procedural protocols are acceptable, such that the conduct under review while novel or unusual may constitute competent practice in the circumstances of the case.^[4-5]^ It is highly recommended to speak to other medical practitioners with similar level of training or experience if it is unclear what the standard of care against which performance should be assessed using this guide. Some important considerations when seeking to assess conduct and competence this way:

- This defence is time-bound, and any reflection must only consider contemporaneous evidence about whether the act(s) or omission(s) is widely accepted as competent professional practice at the time it occurred.
- The reasoning behind expert opinions should also be critically appraised as medical practitioners may disagree, the opinion may be based on inaccurate factual assumptions, based on experiences and training outside of Australia, or the evidence provided by other medical practitioners may be unreasonable or irrational, often referring to conduct so out of step with professional practice.^[6]^
- If there are competing opinions, the evidence relied upon need only show the conduct is widely accepted. No compromise needs to be reached nor do decision-makers need to provide a reason for their preference of one opinion over another.^[7]^
- The opinion(s) must comment specifically on the particular acts or omissions against the totality of circumstances under review.
- Factual inaccuracies underlying peer assessments of conduct and competence often result in the disapplication of the affected opinion. This is key concern when performance data is used as evidence may rely upon inappropriate data analysis methods or incomplete/inaccurate raw datasets.
- This defence is not available for incidents where there was a failure to give warnings, advice or other information with respect to material risks.

Appendix B: Procedural and Substantive Ethical Values

| **Substantive Values** | | | |
| --- | --- | --- | --- |
| Autonomy |  | the ability to freely exercise clinical judgment and expertise when making decisions about self- or team performance. Any intervention from internal and external stakeholders should be clearly justified by best practice or law. | |
|  |  |  | |
| Equity |  | resolve all issues or concerns without discriminating on the basis of personal characteristics or biases while acknowledging disparities in patient populations and the health workforce. | |
|  |  |  | |
| Harm minimisation |  | avoid or reduce harms or the risk of harm to all persons, including the health professional. This includes understanding contributing factors, proposing actions to prevent recurrence and learning lessons from poor or under-performance. | |
|  |  |  | |
| Integrity |  | demonstrate professionalism and good character in all circumstances, including reporting or seeking advice on the disreputable, dishonest, disrespectful or otherwise questionable behaviour of your peers. Any deviations from expectations of your role and responsibilities should be justified to enhance trustworthiness of the health system. | |
|  |  |  | |
| Justice |  | treat all persons fairly with due consideration of interpersonal, organisational and system issues that contribute to performance issues or concerns. Assemble fair arrangements of resources, opportunities and privileges surrounding practice analytics. | |
|  |  |  | |
| Openness |  | explain fully how the evidence collected will be used to monitor and evaluate performance, including the potential benefits and risks for key stakeholders. Being open and honest about this reflection/review and evidence collection will build trust in practice analytics. | |
|  |  |  | |
| Privacy |  | ability to control access, use and disclosure of information/evidence in accordance with values and preferences of patients and the health workforce. Confidentiality and disclosure should be considered alongside privacy as secure and meaningful use of information/ evidence builds confidence in practice analytics and encourages ongoing participation. | |
|  |  |  | |
| Public benefit |  | maximise overall good to the public with full consideration of the health and wellbeing of patients, health workforce and broader health system. The primary purpose of decisions and proposed actions should be to improve the quality and safety of medical services. | |
|  |  |  | |
| Respect |  | care for the dignity and wellbeing of all persons by acting in a compassionate and culturally safe manner. Provide due consideration of their perspectives and demonstrate empathy towards their feelings about the performance/conduct or the reflection/review itself. | |
|  |  |  | |
| Solidarity |  | ensure equitable distribution of opportunities, privileges and burdens to create a safe environment where key stakeholders are able to support each other to optimise performance. | |
|  |  |  | |
| Trust |  | have regard to the value, behaviours and relationship that underpin professional excellence to maintain public confidence in the quality and safety of health services. It also entails cooperation and coordination with key stakeholders to ensure decisions build a constructive learning environment informed by a diverse set of expertise. | |
|  | | |  |

| **Procedural Values** | | |
| --- | --- | --- |
| Accountability |  | Be amenable to review and scrutiny of your performance to encourage openness and build trust in your clinical practice. Understand and accept responsibility for your assigned or actual actions, decisions and their impacts. |
|  |  |  |
| Consistency |  | Similar outcomes for reflections/reviews with similar background facts or issues. |
|  |  |  |
| Efficiency |  | deliver your decision in a timely manner, communicate progress to key stakeholders and be upfront about delays. |
|  |  |  |
| Engagement |  | meaningful participation of key stakeholders involved in or affected by the reflection/review. This includes the right to review information/evidence available to the decision-maker and the right to respond to this information/evidence particularly disagreeable facts. It is a property of natural justice or procedural fairness. |
|  |  |  |
| Honesty |  | be trustful and fair in your speech, actions and relationships. Promote proactive disclosure of actual, or the appearance of, pecuniary or other conflicts of interest to build trust towards routine use of performance data in clinical practice. |
|  |  |  |
| Impartiality |  | an independent decision-maker who could consider the evidence without bias or prejudice. It is a property of natural justice or procedural fairness. |
|  |  |  |
| Proportionality |  | proposed action(s) should be appropriate and adapted to the risk or, harm caused using the minimum force or punitive measures required to protect and uphold patient safety and high-quality care. |
|  |  |  |
| Reasonableness |  | ensure your reflection/review is fair and acceptable to a reasonable person in your shoes, taking into account the perspectives of key stakeholders. Resolve any issues or concerns with full consideration of the totality of information/evidence presented to you, limited only by relevance to the performance/conduct under reflection/review. |
|  |  |  |
| Stewardship |  | secure and standardised management of data assets to prevent unauthorised access to information in accordance with your statutory and professional obligations. This includes a clear outline of responsibilities and accountabilities for each stakeholder accessing or using performance data. |
|  |  |  |
| Transparency |  | be open and honest about your data management, reflection/review procedures and reasons for proposed action(s) to promote regular and constructive communication. This facilitates learning and builds trust with key stakeholders. |
|  | | |

Appendix C: Legal Explainers

These legal explainers provide a brief introduction to legal rules and procedural principles that guide investigations into performance and conduct by professional performance panels, civil and administrative tribunals, and/or the courts. It may be helpful for Framework users to understand the constraints and rigors of fact-finding tribunals and courts of law to ensure a robust and proper consideration of all relevant matters, while appreciating how procedural justice may be instilled into local decision-making. This is not a substitute for legal advice, and you are encouraged to contact a qualified legal practitioner for further information, advice or clarification.

Identify stakeholders and collect information/evidence

**Rules of evidence**

Performance and professional standards panels, performance assessments and state tribunals as administrative or regulatory decision-makers are often not bound by the rules of evidence. This constraint is often excluded from application to the monitoring of, and investigations into, health care performance, health services administration and compliance with privacy and freedom of information laws by statute. This removal is also often accompanied by a mandate to conduct proceedings with as little formality and technicality required to properly consider the matter before the decision-maker. The purpose of removing this requirement is to imbue administrative bodies with sufficient flexibility to collate a wide range of evidence. At the same time, the principles of natural justice, including requirements of procedural fairness, must continue to be observed unless clearly excluded from operation by statute. The content of this duty does vary from matter to matter as it requires the decision-maker to act fairly in support of a fair hearing, but it is often construed as requiring a decision maker to take into account all relevant evidence before them, and give each individual person whose rights and interests may be affected by the decision an opportunity to review and respond to the evidence.^[8]^

While this Framework is designed to be used at a local level before escalation to administrative bodies, the voluntary application of these rules of evidence by these bodies may be helpful to you. Some rules of evidence to consider:

- Ask yourself, could the evidence in question directly or indirectly affect an assessment of the existence of a fact in issue?^[9]^ This is the test of **relevance.** It does not matter if there is a question about authenticity of the documents produced, or even if you decide not to place any significance on this fact in your final decision. If you are unsure, err on the side of including the evidence in your decision-making, as you can still evaluate its authenticity against the totality of information before you.
- Ask yourself, is the person telling you a first-hand account of their experiences (i.e., I saw X do A) or recounting experiences of another person (i.e., Y told me that X did A)? This may be **hearsay** evidence. Generally speaking, the latter should not be considered as we do not know if the person had personal knowledge of the information presented as a fact and therefore if the presented information is indeed true. For Framework users, the tendering of hearsay evidence may indicate you need more information to confirm the facts in issue.
- Ask yourself, why does the person telling you this information say it is relevant to the reflection or review? Is it their own inferences from observed or communicable data? This may be **opinion** evidence. You should consider whether these opinions are necessary to have an accurate representation of the act(s) or omission(s) under review. For example, to understand the impact on patient’s health and quality of life, you may need to collect opinions about their state of mind and emotional state at the time of the act/omission.
- You may even want to collect opinion evidence from experts, as is the case for the application of the peer professional practice when identifying the relevant standard. When relying on expert opinion, ask yourself whether this opinion is based on their training and experience, and whether this training or experience is indeed specialised knowledge or expertise? Have regard to their education, current scope of practice and what facts they relied upon when forming their opinions.^[10]^

**Health data as information/evidence**

You are entitled to use health data you collect for, or in the course of providing, treatment and care as well as employee information for health service evaluation and monitoring for this reflection/review if:

1. patients or health practitioners would reasonably expect this information to be used for this purpose (‘**secondary purpose**’),^[11-2]^ or
2. it is **necessary** to monitor and manage health services,^[13]^ or
3. **express consent** is received from the patient or health practitioner before use in this reflection/review. It is recommended express consent is received for these activities as some jurisdictions require the secondary purpose to be directly related to the primary reason for data collection,^[14-5]^ or prevent use and disclosure of employee information without express consent or a legal order.^[16-7]^

It is recommended to use de-identified information/evidence for routine reflection/reviews. Re-identification of this information/evidence is advisable when a departure from accepted professional standards has been confirmed to inform affected patients, families and health practitioners in the spirit of open disclosure.

Caution is also advised when disclosing personal or health information to regulatory bodies for further investigation. Information/evidence uncovered over the course of the reflection/review relating to the patient or health practitioner that could not be perceived as relevant to the issue or concern may constitute a breach of privacy principles.^[18]^ It is also important here to limit disclosure to the information strictly necessary for the regulatory body or health complaint entity to make a preliminary assessment of the allegation and, if so elected, an investigation into the departure. If unsure, the patient and/or health practitioner should be made aware of this possible disclosure and provided with an opportunity to object to the disclosure of this information. The only clear exception to an alleged violation of patient or practitioner privacy appears to be if the disclosure was necessary to prevent or lessen a serious threat to the life or health of a person or the public.^[13]^

Review and Evaluate Performance

**Reasonable and Reasonableness**

Judicial decisions often consider what a reasonable person in the parties’ shoes would do in the circumstances of the case. It may be helpful to adopt a similar perspective in your reflection/review to:

1. decide whether or not there has been a departure from accepted professional standards;
2. determine the risk of harm and appropriateness of precautions or mitigating action taken;
3. identify areas of improvement and possible actions to improve the safety and quality of care.

Ask yourself:

- Who is the ‘reasonable person’ in the position of the health practitioner and/or patient? Be specific about personal characteristics, experience and scope of practice.
- What is the nature of the activity? It may modify the applicable standard of care, and the ‘reasonable person’ against which the individual’s actions are to be assessed. For example, an unexperienced surgeon who **voluntarily** performs a procedure outside of their scope of practice may be held to the higher standard of a surgeon who is capable of performing the procedure.^[19]^
- How would the reasonable person act in the shoes of the health practitioner? What choices would this reasonable person make? What are the similarities and differences between the hypothetical actions of the reasonable person, and the actual conduct/performance of the health practitioner? Be careful to only consider a reasonable person in **identical circumstances** under review.

This is distinguished from reasonableness as a principle of judicial and administrative review of past decisions.

Specify the issue or concern

**Risk of harm**

Negligence or the ‘failure to exercise reasonable care and skill’ is a common claim in medical litigation. Claimants seek a legal remedy for performance/conduct against persons or organisations that the claimant believes is responsible for performance/conduct that caused the alleged harm or injury. It may be helpful to apply similar considerations in your reflection/review to determine the harm or damage caused by the performance/conduct, or the risk thereof:

- Injury or harm is **reasonably foreseeable.** This test is defined in the negative as in the harm or injury must not be far-fetched or fanciful.^[20]^ It may be helpful to discuss the obviousness of actual or possible injury or harm, incidence or frequency of departure or harm, likelihood of occurrence if precautions were not taken against the risk of this harm, the likely seriousness of the potential or actual harm, the cost and burden of precautions, and whether a reasonable person in the practitioner’s shoes would have taken these precautions.
- The **type** of injury or harm. It could involve physical injuries or damages, emotional distress, a recognised psychiatric illnesses or shock, or economic loss or injury.
- **Request** for advice or information about the experience of their treating practitioner. It may be helpful to consult published data and epidemiological studies about the procedure for information about whether professional experience has a statistically significant impact on the realisation of foreseeable risks. By virtue of performing the procedure, health practitioners make representations to their patients that they have special knowledge and skill in their chosen profession. It may then be the case that an underqualified or less experienced practitioner may be held to be negligent in seeking to perform the procedure without adequate supervision or training. This alleged negligence does not currently create a right to request a specific practitioner perform the procedure in the public health system, or a right to receive performance data about a specific practitioner.^[21]^

These principles are moderated by state-specific legislation on civil liability for negligence. You are encouraged to review these laws prior to taking legal action.

Propose actions and give reasons

**Accountability (or Causation)**

When an action in negligence is brought before the court, the claimant must show the harm is linked to the breach of the duty of care owed by the health practitioner to the claimant. This is known as causation. Legal jurisprudence around causation may be useful when apportioning responsibility for clinical performance and governance, and deciding on an appropriate course of action, possibly including disciplinary measures against the health workforce. Ask yourself:

- Would the departure have eventuated, or the harm or injury occurred, but for the performance/conduct of the health practitioner? This is known as the ‘**but for**’ test.^[22]^ Neither statute nor case law requires the practitioner’s performance/conduct to be the exclusive cause of the departure or harm. It only needs to be a **necessary condition**.^[23-4]^
- Was the harm or risk thereof a “direct and natural”^[25]^ result of the health practitioner’s performance/conduct? **Intention** to depart from professional standards and/or cause injury or harm under review may be useful when deciding if the health practitioner should be held responsible for the departure or harm.
- Were there other causes or contributing factors to the departure? It may be helpful here to consider whether organisational or structural factors contributed to the conditions that resulted in this risk of harm or even the performance/conduct itself, and what changes could be made as a health service to address these non-workforce related conditions.
- Could the patient, other members of the health workforce or the health service have taken their own precautions against the risk of harm? Were these other stakeholders aware, or ought to have known, of the risk?^[26]^ This is known as **contributory negligence** and may be helpful in identifying other stakeholders who may help address the risk moving forward.

References

[1] *Woollard v The Medical Board of Australia Sitting As A Performance and Professional Standards Panel* [2016] WASCA 151 at [42].

[2] *Rogers v Whitaker* [1992] HCA 58.

[3] Mahar PD, Burke JA. What is the value of professional opinion? The current medicolegal application of the “peer professional practice defence” in Australia. *MJA* 2011;194(5):253-5.

[4] *Sparks v Hobson* [2018] HCATrans 191.

[5] *Dean v Pope* [2022] NSWCA 260.

[6] Pakchung D, Smith M, Hughes C. The role of clinical guidelines in establishing competent professional practice. *Aust J Gen Pract* 2019;48(1/2):22-4.

[7] *Polsen v Harrison* [2024] NSWCA 224 at [60].

[8] Robertson A. Natural justice or procedural fairness. Australian Journal of Administrative Law. 2016;23(3):155-63.

[9] *Papakosmas v The Queen* (1999) 196 CLR 297 at [81].

[10] Judicial Commission of New South Wales. Civil Trials Bench Book – [4-0630] Exception: opinions based on specialised knowledge – s 79(1). Available from: <https://www.judcom.nsw.gov.au/publications/benchbks/civil/opinion.html#id-1.7.5.8.11>

[11] *N v Commonwealth Agency* [2009] PrivCmrA 17.

[12] Office of the Australian Information Commissioner, Australian Privacy Principles Guidelines: Privacy Act 1988 (Report, December 2022).

[13] Office of the Australian Information Commissioner, Guide to Health Privacy (Report, September 2019). Available from: [chrome-extension://efaidnbmnnnibpcajpcglclefindmkaj/https://www.oaic.gov.au/__data/assets/pdf_file/0020/251183/Guide-to-Health-Privacy-Collated-May-2025.pdf](https://chrome-extension://efaidnbmnnnibpcajpcglclefindmkaj/https:/www.oaic.gov.au/__data/assets/pdf_file/0020/251183/Guide-to-Health-Privacy-Collated-May-2025.pdf)

[14] *Health Services Act 1988* (VIC).

[15] *Privacy and Personal Information Protection Act 1998* (NSW).

[16] *Health Transparency Act 2019* (QLD).

[17] *Health Services Act 2016* (WA).

[18] *JD v NSW Medical Board* [2005] NSWADT 247.

[19] *McHale v Watson* [1966] HCA 13.

[20] *Wyong Shire Council v Shirt* [1980] HCA 12.

[21] Madden WJ, McIllwraith J, Madden B. Duties regarding informed consent. In: Australian medical liability, 3^rd^ ed. LexisNexis Butterworths; 2017.

[22] *Wallace v Kam* [2013] HCA 19.

[23] *Strong v Woolworths Ltd* [2012] HCA 5 at [20].

[24] *Civil Liability Act 2002* (NSW) s5D(1).

[25] *Palmer Bruyn & Parker Pty Ltd v Parsons* (2001) 208 CLR 388 at [81].

[26] *Civil Liability Act 2002* (NSW) s5R.
