# Supplementary material for: Development of an Ethico-Legal Framework for Quality Improvement and Performance Management in Health Care: Protocol for a Qualitative Study
Source: JMIR Res Protoc. 2026 Jan 30;15:e82167. doi: 10.2196/82167 (PMC12857889; doi:10.2196/82167)
Supplement: Multimedia Appendix 2 [file resprot-v15-e82167-s002.docx]

**Interview/Focus Group Guide**

*[Before recording starts]*

You are here today to review feedback from a series of stakeholder interviews with consumers, health professionals, administrators, regulators and more. This feedback has been synthesised into a core set of principles and legal considerations that should guide decision-making around the use of performance data in clinical practice. In particular, the use of performance data for quality improvement and performance management. This feedback has also been combined with findings from an extensive literature and document review on practice analytics.

You have been provided a copy of this framework in advance of this [interview/focus group], but I will also provide you an opportunity to review it again at the start of this [interview/focus group]. You will then be asked a series of questions about the content, structure and proposed uses of the framework. With your consent, this [interview/focus group] will be recorded. Identifiable information will only be accessible to the interviewer with all data stored on a password-protected file on a secure data store hosted by the University of Sydney. The recording will be transcribed and de-identified by the interviewer before analysis by the wider team.

Q1. Do you have any questions?

- Yes --> *Answer the questions.*
- No ---> *Proceed to next question*

Q2. Are you happy to have our conversation today recorded?

- *Yes -->* I will now turn on the recording function and ask you to confirm your consent for the recording.
- *No -->* Would you be happy for me to take written notes about our discussion?
  - Yes: Record consent and start interview/focus group.
  - No: Inform the participant we will be unable to proceed with the [interview/focus group]. If the participant wants to remain, ask the participant to remain silent throughout the focus group, or seek shorter feedback (maximum of 10 minutes) that will not be used in the project.

*[Start recording]*

Q3. Could you please confirm your consent for the recording?

**Interview/Focus group questions**

***Introductions/Background***

1. Could you please provide a brief introduction (name, organisation and relevant expertise)?

***Framework flow, comprehension and intended audience***

1. Could you please share your initial thoughts or feedback on the framework?
2. Who do you believe is the intended audience of the framework? Who would be the primary users of this type of framework?

Prompts:

- Would this guide work for a health professional reflecting on their own practice and a manager reviewing performance?
- Does it need to be split more clearly into continuous professional development/reflection and performance review? Perhaps into two separate documents?
- Would it work for a relatively inexperienced and experienced health professional or administrator?

***Framework principles: Comprehensiveness, descriptions and relevance***

1. Does the guide provide sufficient guidance to evaluate performance and resolve concerns about professional performance? Why or why not?

Prompts:

- Would it be helpful? Would it be used?
- What’s missing to make the guide actionable?
- Does any prompt feel leading, judgmental or likely to trigger defensiveness?
- Where in the sequence do you expect mistakes or drift from the process?
- Does the step-by-step guide reflect how you would approach performance monitoring and evaluation? Why or why not?

1. Based on your expertise or clinical experience, are the ethical values and/or legal considerations we’ve included relevant and fit for purpose?

Prompts:

- When you read this list of values, what stands out as most important for guiding performance reviews?
- Do any of these values feel less relevant or unnecessary in this context?
- Do the rationales in the appendix reflect your understanding of these principles? Are the values poorly represented?
- What values are missing or is there considerable overlap between values making them redundant?

***Value, Utility and Limitations of the Framework***

1. If this framework was adopted tomorrow, what ethical or legal risks might remain unaddressed? What are other limitations of this framework?
2. What features are needed to make the framework workable in day-to-day practice?

Prompts:

- What resources or information would you need to support its implementation and compliance with the framework?
